# Kind Words for the March Editorial

**Published:** 1894-05

**Authors:** B. L. B. Baylies

**Affiliations:** 418 Putnam Avenue, Brooklyn, N. Y.


### KIND WORDS FOR THE MARCH EDITORIAL.

418 Putnam Avenue,
Brooklyn, N. Y., March 22d, 1894.

Editor of The Homœopathic Physician:

Please find inclosed check to pay my subscription.

Your journal is our staunchest bulwark of the true faith. I am delighted with Dr. Lee's "Warning of Constantine Hering" in the March number, and wish every medical society may be supplied with a copy for each member. I would willingly subscribe something toward that object.

With thanks for your great work,

Yours truly,
B. L. B. Baylies.

